# A capaciflector provides continuous and accurate respiratory rate monitoring for patients at rest and during exercise

**DOI:** 10.1007/s10877-021-00798-7

**Published:** 2022-01-18

**Authors:** Nick Hayward, Mahdi Shaban, James Badger, Isobel Jones, Yang Wei, Daniel Spencer, Stefania Isichei, Martin Knight, James Otto, Gurinder Rayat, Denny Levett, Michael Grocott, Harry Akerman, Neil White

**Affiliations:** 1 Perioperative & Critical Care Theme, Southampton NIHR Biomedical Research Centre, University Hospital Southampton / University of Southampton, Southampton, UK; 2 School of Electronics and Computer Science, University of Southampton, Southampton, UK; 3 Department of Engineering, Nottingham Trent University, Nottingham, UK; 4 Department of Anesthesiology, Duke University School of Medicine, Durham, NC USA

**Keywords:** Respiratory rate, Respiratory monitoring, Critical care, Perioperative medicine, Capaciflector, Sensor

## Abstract

Respiratory rate (RR) is a marker of critical illness, but during hospital care, RR is often inaccurately measured. The capaciflector is a novel sensor that is small, inexpensive, and flexible, thus it has the potential to provide a single-use, real-time RR monitoring device. We evaluated the accuracy of continuous RR measurements by capaciflector hardware both at rest and during exercise. Continuous RR measurements were made with capaciflectors at four chest locations. In healthy subjects (n = 20), RR was compared with strain gauge chest belt recordings during timed breathing and two different body positions at rest. In patients undertaking routine cardiopulmonary exercise testing (CPET, n = 50), RR was compared with pneumotachometer recordings. Comparative RR measurement bias and limits of agreement were calculated and presented in Bland–Altman plots. The capaciflector was shown to provide continuous RR measurements with a bias less than 1 breath per minute (BPM) across four chest locations. Accuracy and continuity of monitoring were upheld even during vigorous CPET exercise, often with narrower limits of agreement than those reported for comparable technologies. We provide a unique clinical demonstration of the capaciflector as an accurate breathing monitor, which may have the potential to become a simple and affordable medical device.

Clinical trial number: NCT03832205 https://clinicaltrials.gov/ct2/show/NCT03832205 registered February 6th, 2019.

## Introduction

Respiratory rate (RR) is an important physiological marker of patient deterioration and it helps to predict mortality risk [1–3]. Specifically, RR elevation is a precursor to intensive care unit admissions [4], cardiac arrest [5], and death [6]. Therefore, early warning scores (EWS) in hospitals include RR to monitor deteriorating patients [7]. However, in recovery rooms and inpatient settings, RR is infrequently measured through bedside counts by observers. This process is time-consuming and often inaccurate [8, 9] due to human error, which can delay urgent clinical actions [1, 10]. For clinicians, RR values below 12 breaths per minute (BPM) may be seen with excess opioids, while higher respiratory rates above 20 BPM may indicate sepsis [2, 11]. In the community, RR is also predictive of patient deterioration with chronic diseases [12]. Recently, the impact of COVID-19 has focused attention towards real-time respiratory rate monitoring [13]. This is becoming an essential requirement for certain ward or ambulatory patients [12] or those taking exercise during clinical cardiopulmonary exercise testing (CPET) [14]. CPET is now often used to evaluate patient fitness and suitability for major surgery in perioperative medicine. Currently, critical care units and operating theatres often rely on capnography or thoracic impedance using ECGs to monitor continuous respiratory rates. Outside these environments, there is no commonly used, non-invasive, accurate, comfortable to wear respiratory rate monitor that has been widely taken up into routine clinical practice.

Presently available RR monitors rely on both established and emerging technologies. For intubated patients or those with a face mask, capnography, spirometry and pneumotachography are frequently employed (reviewed in detail in [14]). For those without ventilatory support, impedance pneumography [15] can provide electrode-mediated RR calculation in still patients, but required electrodes and cables can limit mobility and impede enhanced recovery after surgery (ERAS). A chest belt strain gauge can be more accurate [14] but uncomfortable for long-term monitoring [16]. More novel approaches include depth sensing cameras [17] for remote monitoring and directly attached wearables such as RespiraSense™, which measures thoracic movements through a piezoelectric sensor array [18]. Such emerging technologies identify the technical requirements that non-invasive respiratory monitors must meet, including simplicity, low cost, and accuracy of measurement, both at rest and during movement. Indeed, motion artefacts and limited sensor accuracy during movement are clear technical concerns [19]. So, despite the clinical needs, few existing technologies have yet provided a widely adopted RR monitor in routine clinical practice for awake patients.

**Fig. 1:**
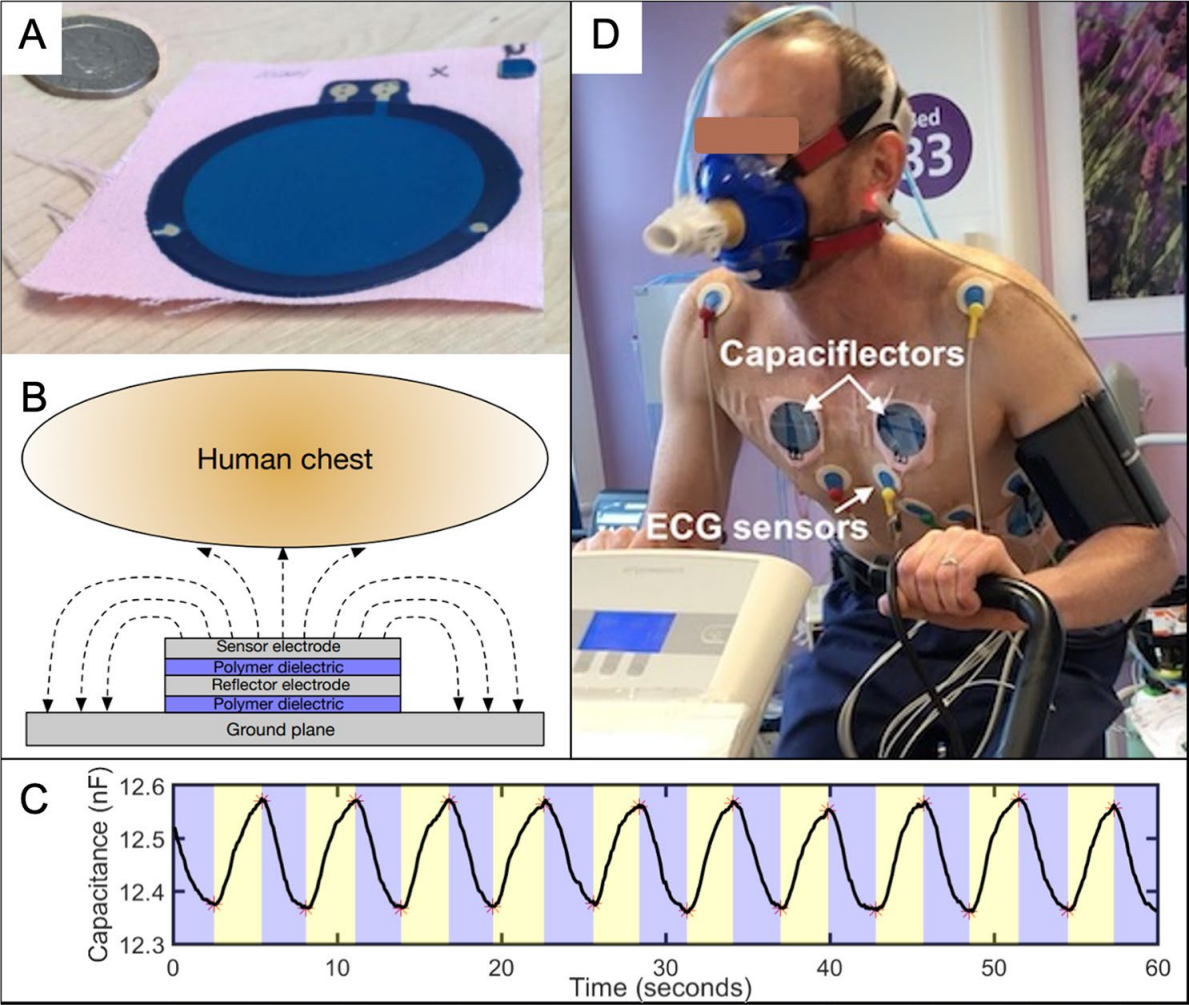
The capaciflector as a respiratory rate (RR) sensor. **A**: Photograph showing one printed capaciflector sensor on fabric, with a 20 pence coin added for scale. **B**: Diagram showing the structure of the capaciflector that detects changes in capacitance as the thorax moves, providing the sensor signal. **C**: Example of the sensor signal (capacitance change) for a 60 s measurement time. The blue and yellow shaded regions indicate exhalation and inhalation, respectively. **D**: Photograph of a healthy volunteer wearing a pneumotachometer mask setup and demonstrating capaciflector placement on the chest during cardiopulmonary exercise testing (CPET), who provided written informed consent for image publication. The ECG dot electrodes are labelled for comparison

The capaciflector has the potential to provide a robust RR monitor [20]. A capaciflector is a proximity sensor based on electrical flux deflection (see [20]). Capaciflectors are thin, flat, flexible sensors that can be attached to patients without skin surface preparation. They are small (a few cm^2^), lightweight (less than ten grams) and can be readily printed at low-cost (Fig. 1). Beyond our prototype, the technology could therefore be developed into a small sticker, or be a sensor within smart textiles. Our novel present study aimed to evaluate the potential for capaciflectors to provide novel non-invasive RR monitoring hardware at rest and during exercise. Based on our understandings, we proposed two hypotheses:

- The capaciflector can measure respiratory rate continuously and accurately, both at rest and during exercise movements.
- Capaciflector hardware accuracy is not influenced by thoracic location or subject position.

Therefore, we designed two studies to test these hypotheses. Study 1: Capaciflector evaluation with healthy subjects at rest, at four chest locations, during normal breathing and metronome timed breathing over a ten-minute period. Study 2: Capaciflector evaluation in patients during cardiopulmonary exercise testing, at the same four chest locations, as part of a clinical trial.

## Materials and methods

### Study 1: an observational comparative study in volunteers

We designed a comparative study of two RR measurement methods: the capaciflector sensor and a chest belt strain gauge monitor. This study included 20 healthy volunteers at the School of Medicine and School of Electronics and Computer Sciences, University of Southampton, UK. Included participants were adults able to give written informed consent in English, physically able to take part, American Society of Anesthesiologists (ASA) grade 1–2 with body mass index (BMI) between 20–30 kg/m^2^. The exclusion criteria were known allergy to medical grade tape, significant chest deformity, implantable defibrillator in situ, spinal cord stimulator in situ, pacemaker in situ and pregnancy. Subjects were provided with a subject information sheet, consent form and questionnaire at time of recruitment. Demographic data were also recorded: age, sex, weight and height. The four capaciflector locations were the left and right precordia (channels 1 and 2), and the left and right axillae (channels 3 and 4), secured using medical grade tape. The participants also wore a chest belt monitor around their torso, positioned and checked to ensure no interference with the capaciflectors. The chest belt monitor (Go Direct ® Respiration Belt, https://www.vernier.com/product/go-direct-respiration-belt/) was connected to a laptop by a USB connection. Data from this device were monitored on Vernier Graphical Analysis (https://www.vernier.com/product/graphical-analysis-4/). Data collection from both devices was simultaneous. Data were collected for two 10-min sessions (one sitting, one lying down) and for another 8 min during a breathing exercise guided by a metronome of pre-determined frequency. The metronome frequency was constant for each subject for all 8 min, but randomly assigned to be between 6–14 BPM across the 20 subjects. For ethical approvals, this study gained Ethics and Research Governance Online approval (ERGO II, Project 56,691) via the Faculty of Medicine Ethics Committee and the Research Integrity and Governance team, University of Southampton, UK.

### Study 2: a clinical observational comparative study in patients undergoing cardiopulmonary exercise testing (CPET)

This study aimed to test capaciflector performance at the same four chest locations as Study 1 but during exercise in a non-targeted sample of preoperative clinical patients presenting for routine CPET before their elective major surgery (see clinicaltrials.gov NCT03832205) within University Hospital Southampton, UK. Fifty of these CPET patients provided their written informed consent to wear four capaciflectors. Capaciflectors were secured by hypoallergenic medical grade tape, in addition to routine CPET monitoring equipment. The four capaciflector locations were the left and right precordia (channels 1 and 2) and the left and right axillae (channels 3 and 4). Raw data were collected throughout each CPET simultaneously with pneumotachometer RR measurements (Ergoflow flow sensor, Geratherm Respiratory GmbH, Germany). The continuous capaciflector and pneumotachometer recordings formed the basis of our data collection for subsequent analyses. Demographic data of age, height and weight were also recorded.

Eligible patients were adults with capacity to provide written informed consent and physically able to undertake their planned, routine CPET. Exclusion criteria for our study were the same as for CPET itself, as previously published by our team [21], with the additional exclusion of those patients with a pacemaker, in situ defibrillator, spinal cord stimulator, or known allergy to medical grade tape.

### Cardiopulmonary exercise testing (CPET) protocol

Patients cycled on an electromagnetically braked ergometer (Ergoline 2000, Ergoline GmbH, Bitz, Baden-Württemberg, Germany). Respiratory gas analysis was performed using calibrated metabolic carts (Geratherm Respiratory GmbH; Love Medical Ltd, Manchester, UK). Breath-by-breath VO_2_ and carbon dioxide output (CO_2_) were recorded, concurrently with minute ventilation, tidal volume, respiratory rate, and end-tidal gas tensions for O_2_ and CO_2_. Patients were connected to appropriate monitoring equipment and rested for an initial 3-min period, thereafter, completing 3 min of unloaded cycling. Subsequently, patients performed a symptom-limited incremental ramp test set to 10–20 W^.^min^−1^ (based on patient weight, and age allowing adjustment for clinical status and current activity levels) to deliver an intended test duration of 8–12 min before volitional exhaustion. Test cessation occurred at patient exhaustion or when the cadence reduced below 40 r.p.m. for more than 30 s despite verbal encouragement. After stopping CPET, patients completed a period of unloaded cycling to ‘cool down’.

Our study design and patient information sheet were built in consultation with CPET patients and CPET physiologists. Ethical and regulatory approvals for our peer-reviewed protocol were sought and obtained via the UK Integrated Research Application System (IRAS, Project ID 251,775), yielding UK Heath Research Authority ethical approval (REC 18/WM/0325). Study sponsorship was provided by the Research and Development Department, University Hospital Southampton, UK. We adhered to strict patient confidentiality, data protection and clinical governance standards throughout, including full data anonymization for all subsequent analyses. All research was performed in accordance with local guidelines and UK ethical guidelines. One healthy volunteer provided written informed consent for their anonymized photograph to feature in this publication.

### Hardware details

A capaciflector is a capacitive sensor that has an additional electrode (a reflector), which directs the electric field into the body. Movement of the chest results in a change in capacitance measured between the sense electrode and ground, and this is integrated in a single, compact sensor. Capaciflectors are powered by a 5 V 10 mA voltage regulator, which was connected to a university-issued laptop via a micro-USB to USB connection. The data from the capaciflectors were collated by LabVIEW, which is a virtual instrument workbench software package developed by National Instruments, Texas, USA. New plastic bags were used in every attachment to keep the sensor clean between subjects. The structure and dimensions of each capaciflector sensor is the same as previously reported [20]. A relaxation oscillator was used to convert a change in capacitance (during respiration) to a change in frequency-based signal that can be more easily measured [20]. The square wave output from the relaxation oscillator was measured and recorded using a Data Acquisition Device (USB-6003, National Instruments, Texas, USA) at 25 ksps (kilo-samples-per-second) per channel. Raw signals were saved to a text file using a customised LabVIEW application. Signals were then processed offline using a custom analysis script written in MATLAB 2019b (Mathworks, MA, USA). Briefly, a high pass filter (0.02 Hz cut-off frequency) was used to remove the DC level and any low-frequency noises followed by a low pass filter (1 Hz cut-off frequency), which smooths the signal and removes unwanted higher frequency signals due to movement and other artifacts. The filtered signal was then converted into the frequency domain using a short-time Fourier transformation (sampled at 10 Hz with a 60 s window, and an overlap of 90%). This gives a final resolution of six seconds per measurement point, which was averaged each minute to give the reported respiration rate in breaths per minute.

The capaciflector sensor was compared with a commercially available belt sensor (Go Direct® Respiration Belt, Vernier, OR, USA) in Study 1. The belt sensor was mounted on the chest and measured the force due to chest expansion, which varies during a breath cycle. The sensor was operated as per the manufacturer’s guidelines. The raw data for the force from the commercial belt were recorded at 10 Hz and processed using the same analysis script as for the capaciflector data. For Study 2, the pneumotachometer measured the time a peak in airflow was detected and recorded the time of this event to the nearest second in a text file. These data were converted to a breathing rate by determining the number of breathing events within 60-s intervals. Synchronization for the start time between the capaciflector and reference sensor (pneumotachometer or strain gauge belt) was performed manually within less than a single breathing cycle in all experiments, equating to < 0.5 s (maximum 5% error). Both approaches relied on raw, chest movement data, with resolution beyond that provided by manual observer counts. A pneumotachometer setup was only available in Study 2, in the clinical CPET setting.

**Fig. 2:**
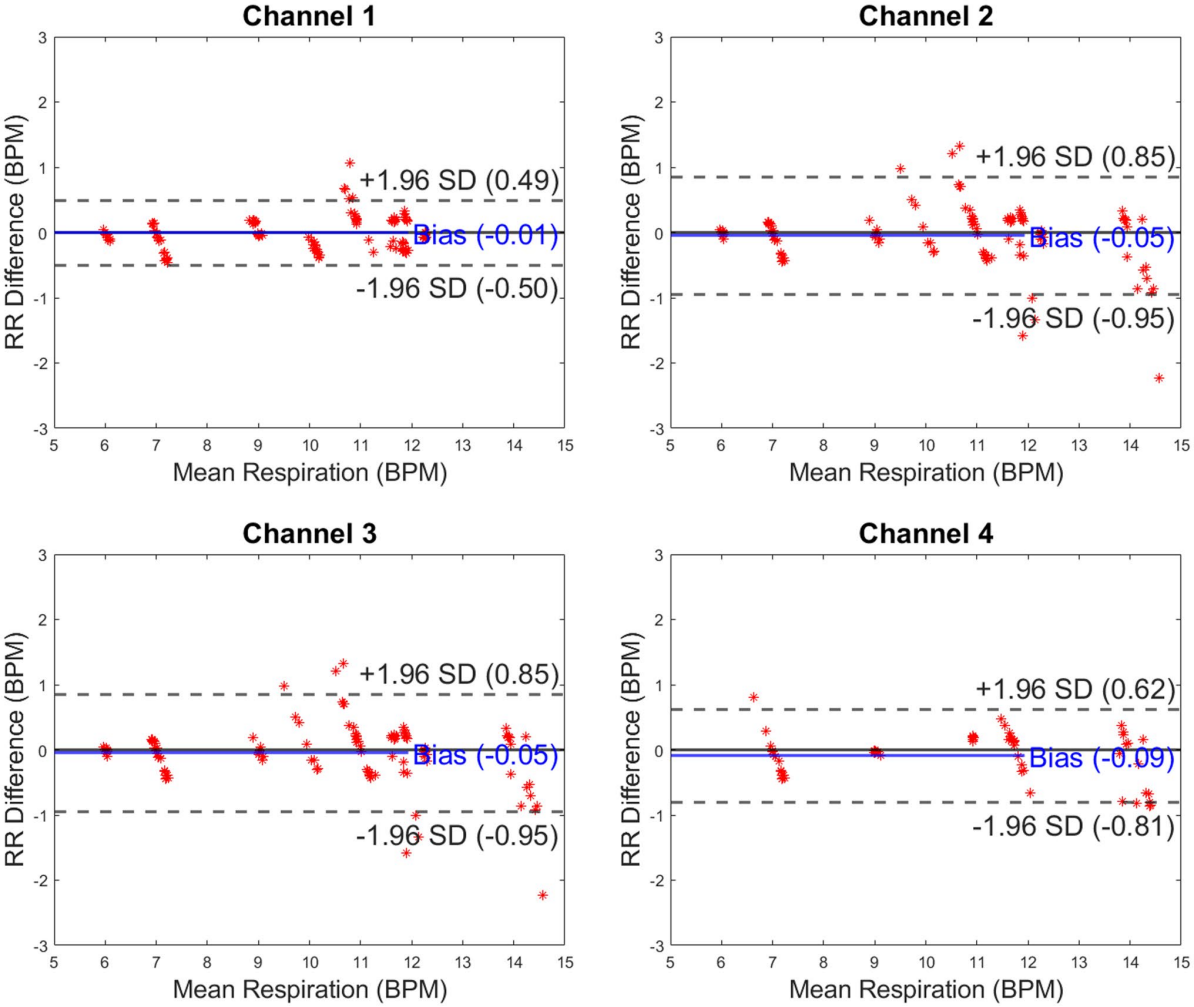
Bland–Altman plots presented by capaciflector channel location during the metronome breathing pattern test for healthy subjects (n = 15, 17, 15 and 8 for channels 1–4, respectively). The comparator was a strain gauge chest belt (Study 1). RR, respiratory rate; BPM, breaths per minute

**Fig. 3:**
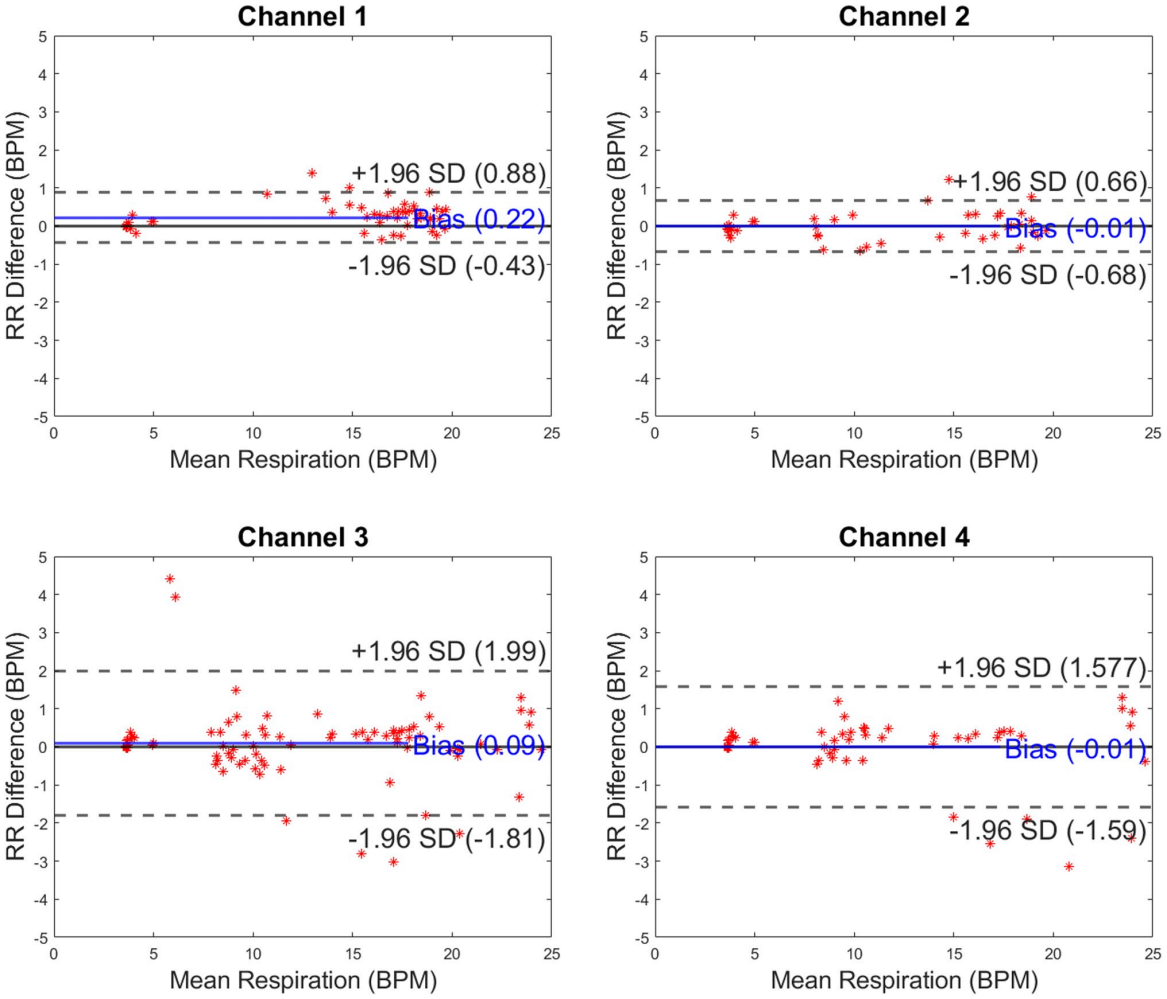
Bland–Altman plots presented by capaciflector channel location while subjects (n = 6, 6, 9 and 6 for channels 1–4, respectively) were lying down. The comparator was a strain gauge chest belt (Study 1). RR, respiratory rate; BPM, breaths per minute

**Fig. 4:**
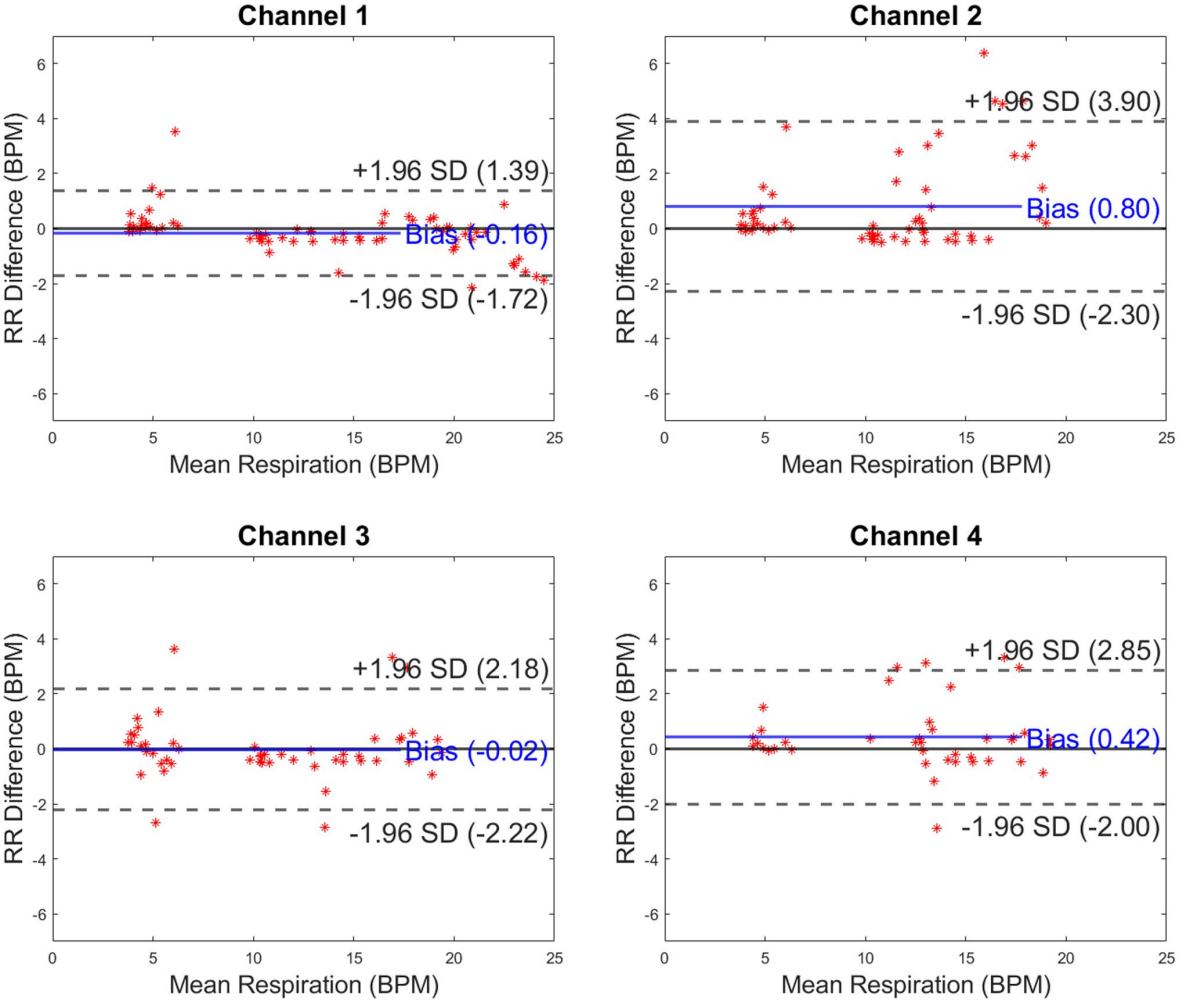
Bland–Altman plots presented by capaciflector channel location while subjects (n = 7, 6, 6 and 4 for channels 1–4, respectively) were seated. The comparator was a strain gauge chest belt (Study 1). RR, respiratory rate; BPM, breaths per minute

**Fig. 5:**
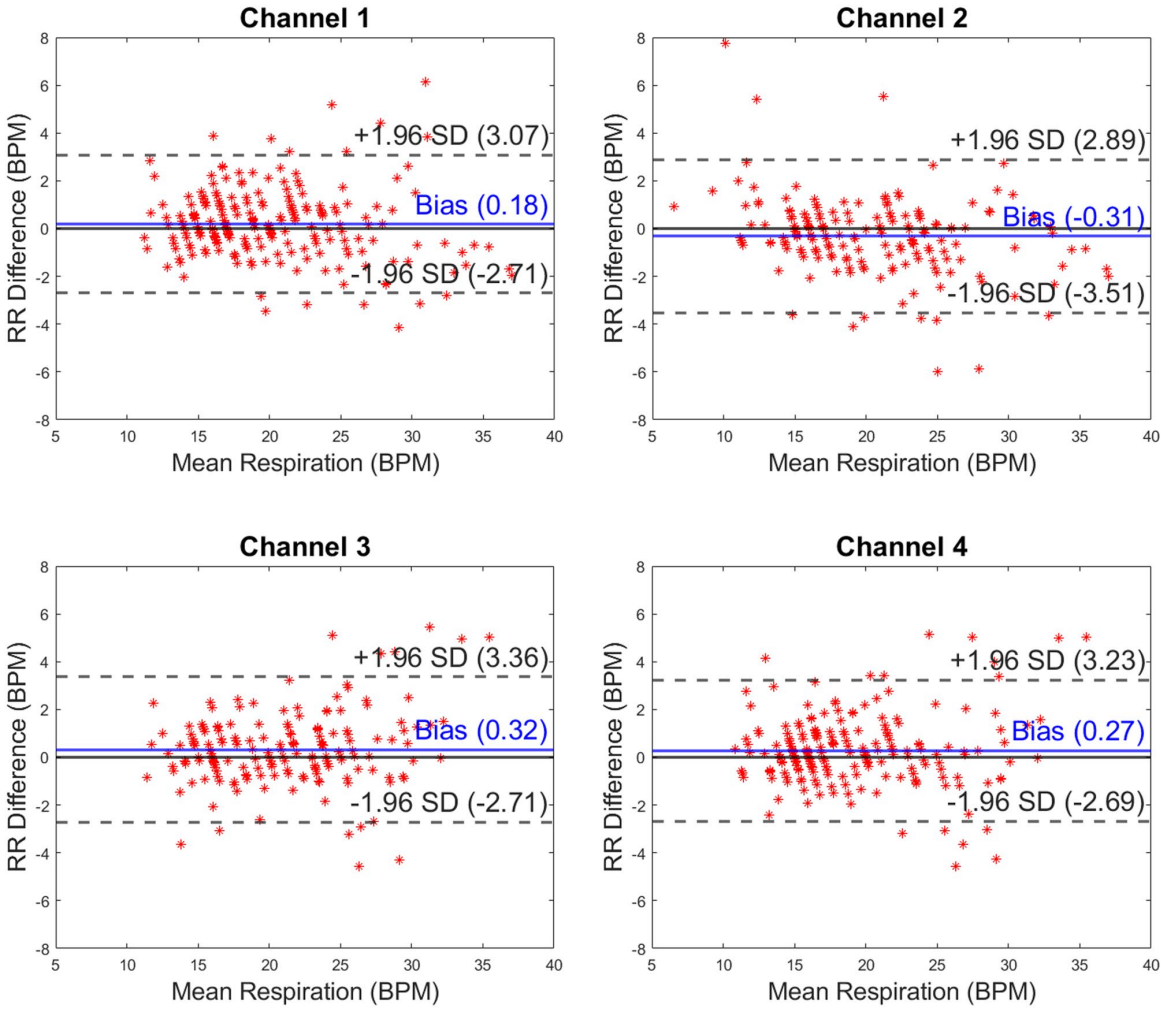
Bland–Altman plots presented by capaciflector channel location while subjects (n = 22, 18, 18 and 20 for channels 1–4, respectively) underwent cardiopulmonary exercise testing (CPET) on an exercise bike. The comparator was a pneumotachometer (Study 2). RR, respiratory rate; BPM, breaths per minute

All usable capaciflector data were included in this study. Owing to the prototype hardware nature of the capaciflector system, some sensor faults were detected, but these were not apparent until the study had concluded because data were processed offline in a blinded fashion. Although the sensors could, in principle, be single use owing to the low fabrication cost, we reused the same sensors throughout Study 1 and Study 2 to maximize the use of the limited numbers available to us. We observed a trend in deterioration as the research progressed, possibly due to moisture in the insulating layers that increases conduction between the electrodes and provides a low-impedance route for the electric field. Data were excluded systematically using the following criteria: (1) a baseline oscillation frequency outside a range of ± 25% of the nominal baseline frequency of 3.6 kHz (typically faulty sensors had an oscillation frequency > threefold higher than the nominal baseline frequency) and (2) > 10 artifacts per minute, where an artifact is a spike in the data that is tenfold higher than the surrounding 10 peaks. Please see figure legends for respective final dataset sizes. Comparisons between capaciflector data and either the chest belt data (Study 1) or pneumotachometer data (Study 2) were made as Bland–Altman plots created in MATLAB 2019b. After inclusion and exclusion criteria were applied, the remaining capaciflector datasets for each channel in each setting were included for the Bland–Altman plots (Fig. 2, Fig. 3, Fig. 4 and Fig. 5). The Bland–Altman method calculates the mean difference between two methods of measurement (the ‘bias’), and 95% limits of agreement as the mean difference (1.96 SD). It is expected that the 95% limits include 95% of differences between the two measurement methods.

## Results

For Study 1, we recruited and included 20 healthy volunteers (11 female) aged 18 to 24 years (mean 20.05 years) with BMI range 20.07 to 29.74 kg/m^2^ (mean 23.51 kg/m^2^). We compared RR measurements between the chest belt sensor and four capaciflectors, providing a Bland–Altman plot for each channel. For the metronome test, across all four channels, the measurement bias provided the difference between recording methods. This ranged between -0.09 to -0.01 BPM (n = 15, 17, 15 and 8 for channels 1–4, respectively), showing that channel RR measurements were comparable between channels within 1 BPM (Fig. 2). The limits of agreement ranged from -0.95 to 0.85 BPM. For the lying down test, across all four channels (n = 6, 6, 9 and 6 for channels 1–4, respectively), the measurement bias ranged between -0.01 to 0.22 BPM. This shows that channel RR measurements were comparable between channels within 1 BPM (Fig. 3). The limits of agreement ranged from -1.81 to 1.99 BPM. For the sitting test, across all four channels (n = 7, 6, 6 and 4 for channels 1–4, respectively), the measurement bias ranged between -0.16 to 0.80 BPM. This shows that channel RR measurements were comparable between channels within 1 BPM (Fig. 4). The limits of agreement ranged from -2.30 to 3.90 BPM.

For Study 2, we recruited and included 50 patients (26 female) during their planned CPET. Participants were aged between 30 to 84 years (mean 65.24 years) with BMI range 18.32 to 50.24 kg/m^2^ (mean 28.28 kg/m^2^). For this study, across all four channels (n = 22, 18, 18 and 20 for channels 1–4, respectively), the measurement bias ranged between -0.31 to 0.32 BPM. This shows that channel RR measurements were comparable between channels within 1 BPM (Fig. 5). The limits of agreement ranged from -3.51 to 3.36 BPM.

## Discussion

In this research, we have shown that the capaciflector measured RR continuously and accurately, both at rest and during exercise. To the best of our knowledge, this is the first clinical demonstration of the capaciflector as a respiratory monitor in patients. We trialed capaciflectors upon patients undergoing CPET so that we could evaluate the sensors across a wide respiratory rate range (rest through to maximal exercise) and under challenging movement conditions, all within a short timeframe. Capaciflector-based RR measurements were comparable with their reference method measurements at each thoracic location and with every subject position in both studies. We note that some capaciflector data for each sensor were lost due to prototype hardware issues. However, via systematic evaluation of the integrity of signal data before all analyses, we were still able to generate valid RR results through sufficient comparisons with the reference methods. Four thoracic sensor positions were chosen to demonstrate that the capaciflector hardware accuracy was not influenced by thoracic location.

As body movement increased between test conditions through Study 1 and with exercise in Study 2, the limits of agreement between RR recording methods broadened. This demonstrates that motion of subjects can impact on the accuracy of RR measurements. In the metronome test results, the mean difference in respiration rate (bias) between the chest belt and capaciflector sensors was minimal, with a bias that was less than 0.1 breaths per minute for all channels (Fig. 2). The limits of agreement were also less than 1 breath per minute for the metronome-directed breathing test. The limits of agreement were broader in the lying down tests for channels 3 & 4 (mounted on the left and right axillae) compared to channels 1 and 2, which were mounted on the left and right precordia. These different locations likely experience different movement directions that give rise to artifacts in the capaciflector signal. Furthermore, the tidal volume will be low especially for healthy young subjects when lying down and resting, resulting in a weaker respiration signal. The low value of bias indicates that on average breaths were not missed or overcounted. The sit test results have a higher limit of agreement compared with the lie test results, which are promising when compared to competing clinical technologies validated in the literature (Table 1). General movements when sitting were uncorrelated with respiration, which sometimes resulted in spurious signals giving a higher breath count (positive RR difference) and sometimes baseline level changes resulting in a missed breath (negative RR difference). Furthermore, we cannot eliminate the chest belt as a source of error. In Study 2, participants wore the capaciflectors while cycling vigorously, which resulted in a higher deviation between the respiration rate determined by the capaciflector and pneumotachometer. This can be attributed to the oscillatory motion during cycling at a similar frequency to respiration rate. All four channels had a similar limit of agreement, i.e. they all have similar motion artifacts. This is expected since cycling creates whole body movements. Nonetheless, the low bias (within one breath/minute) demonstrates no significant over- or undercounting during high levels of exercise movement. This is also within clinically acceptable limits for such monitors [22].

**Table 1:**
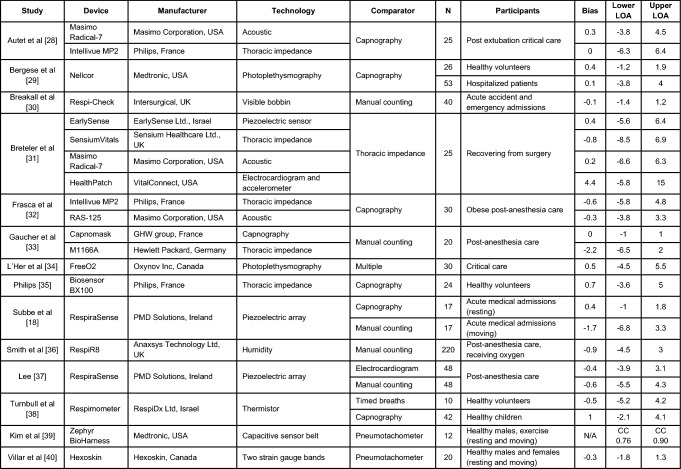
An overview of accuracy investigations of commercially available respiratory rate monitoring technologies

*N*: Number of subjects, *LOA*: Limits of agreement, *CC*: Correlation coefficient (Bland Altman comparison not published), *N/A*: Not applicable

There are several technologies available for measuring respiratory rate (Table 1). Studies investigating these devices are highly heterogenous in terms of the type of device, comparator and study population (clinical/non clinical). We used different comparators because Study 1 was undertaken with volunteers at the University and Study 2 on patients at our neighboring University Hospital. No pneumotachometer setup was available to us at the University. At rest, people tend to breathe differently when wearing a mask as opposed to a body-worn system and we required the tests in Study 1 to be as close to ‘natural’ breathing as possible. The Go Direct device provides a non-invasive, direct measure of chest movement and is hence equivocal to manual chest movement counts. The set-up process does require the belt to be tightly fitted around the chest and is hence not comfortable for long periods. In all cases, the respiration signal from the raw data was strong with no detectable artefacts and hence we believe it provided an excellent comparator device.

Manual counting is one of the most common comparators, but studies have shown both interobserver bias and differences relating to the time interval over which breaths are counted [23, 24]. The accuracies of respiratory rate technologies, especially those based on expansion of the chest, are impeded by body motion artifacts [25]. The majority of existing studies are in subjects who are moving very little, whereas we explored the accuracy of the capaciflector during different body positions, stillness, timed breathing and vigorous exercise. While exercise affected the limits of agreement, it introduced very little bias (Fig. 2, Fig. 3, Fig. 4 and Fig. 5). A comparable thoracic expansion-based sensor has been shown to have a bias (limits of agreement) of 0.38 (1–1.8) at rest, and -1.72 (-6.8–3.3) during movement [18]. The capaciflector was therefore shown to provide a suitable accuracy range in all settings tested in the present study. Such surveillance strategies allow for earlier and more reliable identification of patient deterioration, increased rapid response activation, and lower requirements for patient rescue [26, 27].

We have previously published the theory of how the capaciflector detects chest wall expansion and collapse [20]. During the present research, sensor placement was simple and successful for all 70 subjects, largely due to the lightweight, flat, flexible nature of the sensor. The sensor pads can be readily mass produced through printing, paving the way for a clean, self-adhesive single use sensor in clinical settings. Both the sensors and the conventional electronics that attach to these sensors are therefore amenable to mass-production, and hence they potentially offer an inexpensive sensing solution.

In this research, electrical connections between the capaciflector sensors and our recording setup were sometimes lost due to fragile wiring. As data were analyzed post-recording, loose hardware connections and sensor issues were only identified retrospectively. This allowed data collection to be made in a blinded fashion, yet it did not permit us to identify hardware issues at the time of recording. We witnessed a progressive trend in hardware deterioration, possibly due to moisture in the printed insulating layers, despite each sensor being covered in new clean plastic for every use. However, the acquired data were sufficient for our results and conclusions. We plan to upgrade our prototype hardware through subsequent research.

A key limitation of the capaciflector highlighted through our research is the extensive use of cables, which were required to connect each sensor to the host computer. We are currently developing a wire-free solution that allows both data logging and remote transmission. Power to the circuitry will be provided by a standard coin cell battery capable of providing several days of continuous usage. Further improvements will include compensation for body movement, sweat and variations in temperature, which can also affect the accuracy of the comparator measurement methods as well. Extensive public and patient involvement will be carried out in the coming months to maximize comfort, wearability, and ease-of-use of the device with a view to gaining appropriate medical certification. Evaluation across a range of subject body types and skin types will also be required.

The use of wearables to monitor physiology both in consumer health (Apple Watch/Fitbit) and health conditions is rapidly increasing. For example, diabetic patients use blood glucose readings to control insulin infusion pumps, providing a more physiologically accurate way to manage diabetes than intermittent finger prick readings and bolus insulin injections. Much like abnormal blood glucose alerts coming from diabetic monitors, continuous respiratory rate monitors can alert clinicians and patients to concerning trends and absolute measurements earlier than infrequent manual RR counts [8]. For a new physiological monitor to succeed in healthcare settings, Norman describes a requirement for success in three domains; technology efficacy, marketing triumph, and impressive user experience [16]. Therefore, even when the technology of a new sensor may work, the clinical product may fail to be adopted if inadequately marketed and not acceptable to patients and clinicians. These factors, along with inadequate perceptions of new value, are likely to be the reasons that we do not currently have a commonly used continuous respiratory rate monitor for awake patients. Meng and co-workers identified eleven critical user requirements that wearable healthcare systems should satisfy. Of clinical note, patients rightly expect our devices to have been validated, work properly in their use case, and carry security of data transfer [19]. This means that patient and clinician views on needs such as wearability, ease of set up and use, and the device battery life will all be crucial to make a successful overall product [19]. Similarly, the integration of continuous RR measurements with existing early warning systems in routine use will require careful consideration and validation. A capaciflector, if validated, has the potential to provide such continuous, real-time monitoring of RR and enable sooner recognition of early illness. Therefore, the capaciflector could become a cost effective, safe, single use monitor, which provides many advantages over existing technologies.

## Data Availability

Raw signal data and source code carry intellectual property belonging to the University of Southampton, UK, thus cannot be made available. Processed data for our Figures will be made available at:https://springernature.figshare.com/submit#/s/af1aebf2344224a77d95da52af3ee9ea0f9db3c6c4566cf073ceee5e6ec30028
